# Synergistically Enhanced Properties of Polyimide Films via In-Situ Hollow SiO_2_ Nanospheres

**DOI:** 10.3390/molecules31193567

**Published:** 2026-10-07

**Authors:** Zheng Wu, Rui Meng, Chonggui Zhong, Yanyan Huang, Juehong Guo, Yuchi Cheng, Zhongwei Yu

**Affiliations:** 1 School of Physical Science and Technology, Nantong University, Nantong 226019, China; wuz@boliantech.com (Z.W.); 17851870775@163.com (R.M.); chgzhong@ntu.edu.cn (C.Z.); yyhuang@ntu.edu.cn (Y.H.); 2 Nantong Bolian Tech Co., Ltd., Nantong 226019, China; tech@boliantech.com

**Keywords:** polyimide, hollow SiO_2_ particles, dielectric constant, performance

## Abstract

Polyimide (PI) films serving as high-temperature dielectric and structural materials are increasingly required to combine mechanical robustness, thermal stability, and low dielectric properties for advanced electronic packaging. Herein, PI composite films incorporated with 110 nm hollow SiO_2_ nanospheres were fabricated via in-situ synthesis and subsequent thermal imidization. The optimal comprehensive performance was achieved at a SiO_2_ filler loading of 10%. Relative to pristine PI, the optimal composite delivered a 33% enhancement in room-temperature tensile strength (reaching 68 MPa), a 28% increase at 200 °C (reaching 64 MPa), and a 64% improvement at 250 °C (reaching 66 MPa). Benefiting from the hollow filler structure, this sample also exhibited enhanced ductility (reflected by a 1.3% rise in elongation at break), the most favorable thermal stability among all specimens, and a 3.5% reduction in relative dielectric constant, collectively achieving the synergistic enhancement of mechanical, thermal, and dielectric properties and rendering the composite a promising candidate for high-temperature electronic insulation applications.

## 1. Introduction

Polymers have found extensive applications across diverse technological sectors, benefiting from their tailorable structures and properties, favorable processability and moldability, as well as excellent physicochemical characteristics [1,2,3,4,5]. Among polymeric materials, polyimide (PI) stands out for its promising dielectric performance, outstanding mechanical robustness, and exceptional thermal stability [6,7,8], and it has been widely deployed in high-end fields such as flexible electronics, communication systems, and aerospace engineering [9,10,11,12].

Nevertheless, pristine PI exhibits a relatively high relative permittivity (εᵣ) of approximately 3.5 [10], which cannot satisfy the stringent requirements for low relative permittivity and robust mechanical properties in high-frequency communication, high-density packaging, and other cutting-edge application scenarios [13,14,15]. Accordingly, developing PI nanocomposites with integrated low relative permittivity and excellent mechanical performance has emerged as a topic of intense research interest in recent years [16,17,18,19,20].

Hollow silica (SiO_2_) is a class of silica materials with a hollow interior architecture. Owing to the embedded internal air voids, its relative permittivity can be tuned from 1.1 to 3.0 depending on the internal void fraction, a value substantially lower than that of pristine PI films [21]. Incorporating hollow SiO_2_ nanospheres (HSNs) as fillers into a PI matrix can effectively reduce the relative permittivity of the resulting nanocomposite films, while the intrinsic rigidity of SiO_2_ nanoparticles can also improve the mechanical properties of PI films to a certain extent. The internal void fraction of HSNs, along with their homogeneous dispersion and optimal filler loading in the film matrix, represent critical factors governing the scalable manufacturing of low-permittivity PI films.

To date, most academic investigations into reducing the relative permittivity of PI via filler incorporation have mainly concentrated on blending modification, filler size optimization, and dispersion process improvement. For instance, Abdullaev et al. [22,23] fabricated PI/PTFE composite films via a facile electrospinning strategy combined with amidation reaction, yielding films with relative permittivities of 1.64–1.99 at 10 GHz and an ultralow average relative permittivity of 1.22–1.27 under high-frequency conditions. Zhang et al. [24] prepared SiO_2_/PI composite membranes using self-assembled hollow SiO_2_ tubes, achieving a reduced relative permittivity of approximately 2.9 at 1 kHz.

Nevertheless, systematic investigations into the synergistic correlation between filler loading and the dielectric, mechanical, and thermal properties at a fixed particle size remain limited. In particular, for hollow SiO_2_ with a specific particle size, the optimal filler loading range to achieve a balance between permittivity reduction and comprehensive performance in a PI matrix has not been clearly identified, which hinders the large-scale application of such composite materials.

Against this backdrop, this work employs hollow SiO_2_ with a fixed particle size of 110 nm as the filler and systematically explores its optimal filler loading range in the PI matrix. The findings of this work are expected to provide critical parametric basis and experimental guidance for the practical fabrication and engineering application of low-permittivity PI nanocomposite films.

## 2. Results

### 2.1. Fourier-Transform Infrared Spectroscopy Characterization

**Figure 1:**
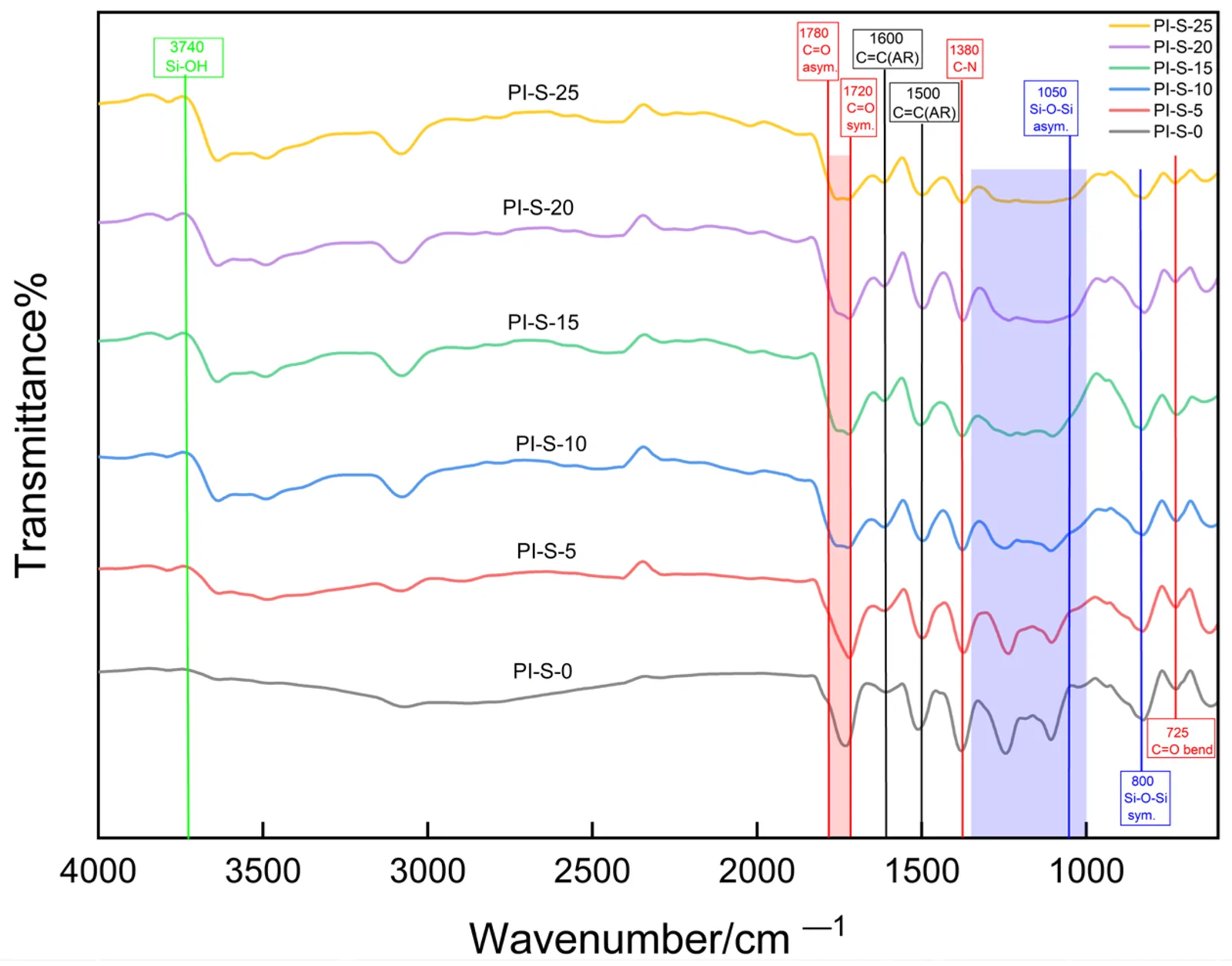
Thin-film FTIR curve.

Figure 1 shows the Fourier-transform infrared (FTIR) spectra of the composite films.

Figure 1 presents the Fourier-transform infrared (FTIR) spectra of PI/SiO_2_ composite films recorded over the range of 4000–600 cm^−1^, with transmittance (%) plotted on the ordinate. Six spectra are displayed, offset vertically for clarity, and are designated from top to bottom as PI-S-25, PI-S-20, PI-S-15, PI-S-10, PI-S-5, and PI-S-0.

Four characteristic imide bands are clearly observed in the spectra, corresponding to C=O asymmetric stretching at 1780 cm^−1^ (Imide I), C=O symmetric stretching at 1720 cm^−1^ (Imide I), C–N stretching at 1380 cm^−1^ (Imide II), and C=O out-of-plane bending at 725 cm^−1^ (Imide IV). Meanwhile, none of the diagnostic absorptions of the poly(amic acid) (PAA) precursor—namely, amide C=O stretching at 1650 cm^−1^, amide N–H bending at 1550 cm^−1^, and the broad N–H stretching band at 3200–3400 cm^−1^—are detected. These results confirm that thermal imidization proceeded to completion, with the PAA precursor fully converted to PI via dehydrative cyclization, and that the incorporation of SiO_2_ did not adversely affect the imidization process.

The characteristic bands of SiO_2_ emerge at 1050 cm^−1^ and 800 cm^−1^. In region (a) of Figure 1 (1000–1350 cm^−1^), unfilled PI exhibits only its own weak, sharp skeletal bands in this range; however, a pronounced evolution of the peak profile is observed within 1000–1350 cm^−1^ as the SiO_2_ loading increases. The underlying reason is that the Si–O–Si stretching vibration is inherently broad: as the loading rises from 5 to 25 wt%, a broad absorption band centered at 1050 cm^−1^ gradually emerges and progressively intensifies and widens, extending toward 1150 cm^−1^ at higher wavenumbers. The PI skeletal band near 1170 cm^−1^, clearly resolved in unfilled PI, becomes increasingly obscured with rising loading. This does not signify the disappearance of the band; rather, it is progressively masked by the growing Si–O–Si broad band via spectral overlap, whereby the band trough is filled and the contrast is diminished. This effect does not indicate any alteration of the PI structure.

A downward bend is observed near 3740 cm^−1^ in the composite films, which is absent in unfilled PI. This feature arises because the O–H stretching vibrations of incompletely condensed silanol (Si–OH) groups on the SiO_2_ surface span the range 3200–3700 cm^−1^, with isolated (free) Si–OH groups appearing at ~3740 cm^−1^. Owing to their strong polarity and hygroscopicity, Si–OH groups readily adsorb atmospheric H_2_O, whose O–H stretching vibration gives a broad band at ~3400 cm^−1^ that overlaps with the Si–OH absorption. As the SiO_2_ loading increases, the total amount of SiO_2_ and the total number of Si–OH groups both rise, enhancing the water-adsorption capacity and thereby intensifying the O–H absorption and deepening the bend. Unfilled PI is nearly featureless in this region. This dose-dependent behavior serves as indirect evidence for the successful incorporation of SiO_2_ into the films.

In summary, four characteristic imide bands (1780, 1720, 1380, and 725 cm^−1^) and two diagnostic SiO_2_ bands (1050 and 800 cm^−1^) are clearly observed in Figure 1. Meanwhile, the PI matrix undergoes no degradation or side reactions upon doping. These findings collectively confirm that SiO_2_ has been successfully and uniformly incorporated into the PI/SiO_2_ composite films.

### 2.2. Mechanical Performance Analysis

**Figure 2:**
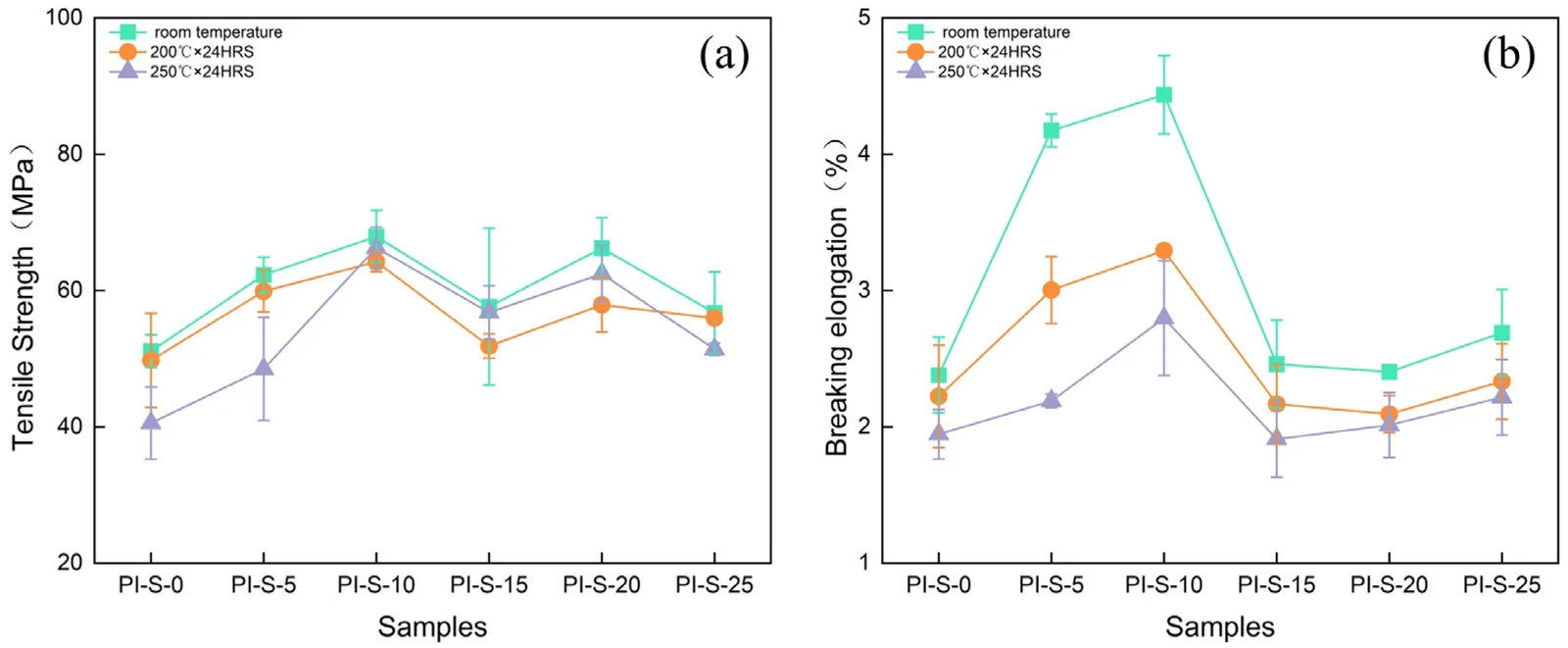
Film mechanical performance testing. (**a**) Tensile strength of the thin films; (**b**) Breaking elongation of the films.

The mechanical properties of composite films exert a crucial influence on their practical applications. Considering the actual service requirements, the mechanical performances of the composite films were tested under three different temperature conditions: at room temperature, cooling to room temperature after thermal treatment at 200 °C for 24 h, and cooling to room temperature after thermal treatment at 250 °C for 24 h. The tensile strength and elongation at break were recorded, and the relevant results are shown in Figure 2. Each sample was subjected to three independent measurements, and the average value was taken as the final result. Detailed data can be found in Table S3 (Tensile strength test data) and Table S4 (Elongation at break test data).

As shown in Figure 2a, the tensile strength presents a non-monotonic fluctuation trend. The composite films exhibit a consistent variation rule at different temperatures: the tensile strength first increases, then decreases, and declines further under all temperature conditions. The three curves share the same peak positions, and double peaks of tensile strength appear at PI-S-10 and PI-S-20. The maximum tensile strength values are obtained at PI-S-10, which are 68 MPa at room temperature, 64 MPa after heat treatment at 200 °C, and 66 MPa after heat treatment at 250 °C, respectively. It can be observed from Figure 2b that the elongation at break increases first and then decreases at all tested temperatures. All samples reach their maximum elongation at break at PI-S-10, with values of 4.5% at room temperature, 3.2% after heat treatment at 200 °C, and 2.8% after heat treatment at 250 °C.

### 2.3. Electron Microscopy Characterization

Transmission electron microscopy (TEM) was employed to characterize the microstructure of hollow SiO_2_ particles. Considering the outstanding mechanical performance of PI-S-10, supplementary scanning electron microscopy (SEM) characterization was further conducted on the PI-S-10 composite film.

**Figure 3:**
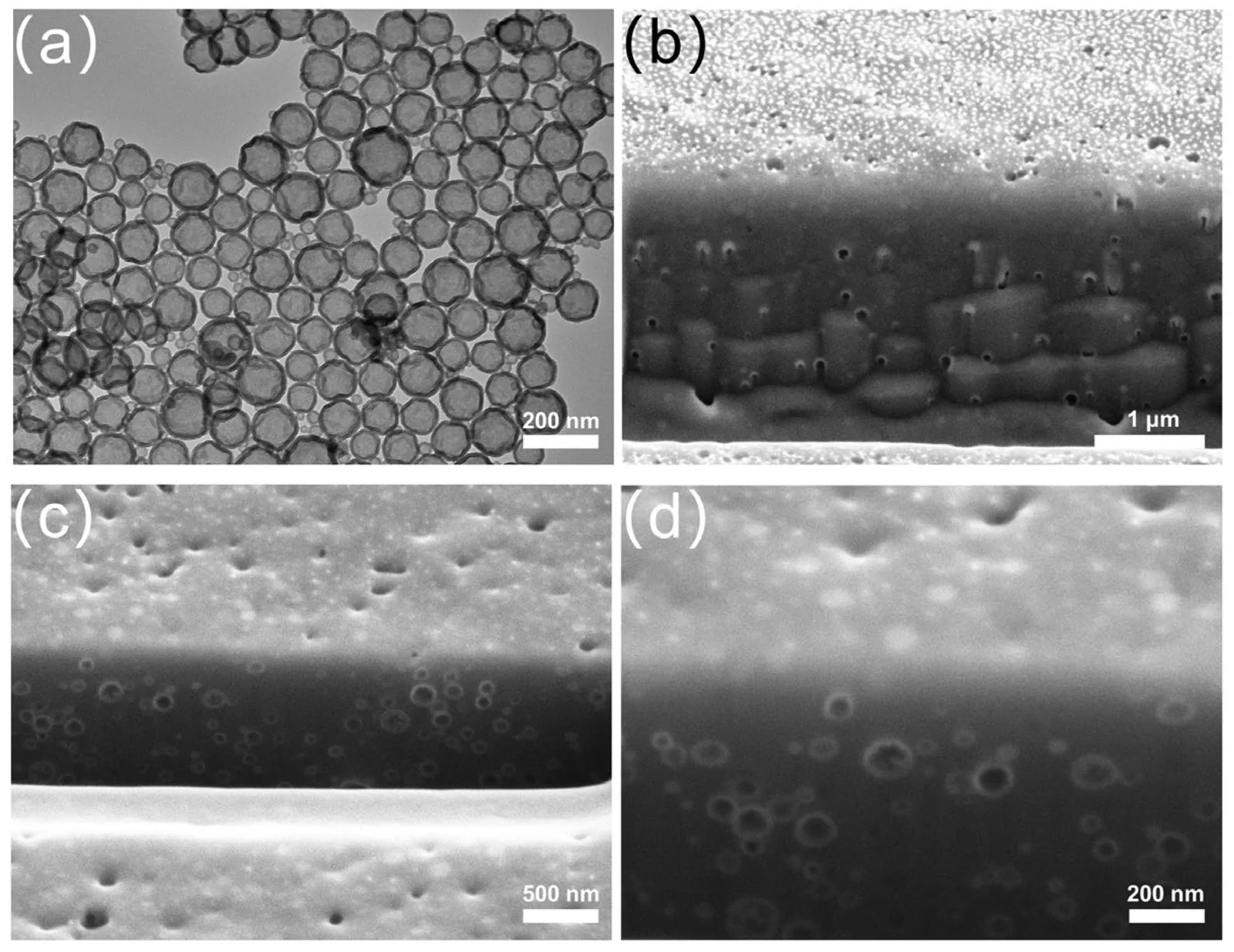
Film mechanical performance testing. (**a**) TEM images of 110 nm hollow SiO_2_; (**b**) high-magnification cross-sectional SEM image of PI-S-10; (**c**) surface SEM image of PI-S-10; (**d**) local magnified surface SEM image of PI-S-10.

It can be observed from Figure 3a that the SiO_2_ particles present a regular spherical morphology. A bright shell layer is formed at the particle periphery with a dark hollow area in the center, which clearly reveals the intrinsic hollow structure of the particles. The particle size of most samples is approximately 110 nm. Figure 3b–d shows the SEM images of the PI-S-10 composite film, and the grooves on the surface are induced by laser treatment. From Figure 3b, it is found that the surface of PI-S-10 is covered with fine and evenly distributed SiO_2_ particles. The cross-section maintains a relatively dense structure with only a small amount of large-sized pores. As displayed in Figure 3c,d, uniformly distributed shallow pits can be observed on the surface of PI-S-10, which correspond to the embedded hollow SiO_2_ particles with a diameter of 110 nm. The filler particles exhibit excellent dispersion without obvious agglomeration, voids or cracks. Moreover, tight interfacial adhesion is formed between SiO_2_ particles and the PI matrix, and no obvious structural defects can be observed.

### 2.4. Dielectric Properties Analysis

Combined with the mechanical performance results presented in Section 2.2, the mechanical properties of composite films begin to deteriorate when the doping content increases to 25%, which can no longer meet the requirements of some practical application scenarios. Therefore, the dielectric constant tests of composite films were only carried out up to a doping content of 25%.

**Figure 4:**
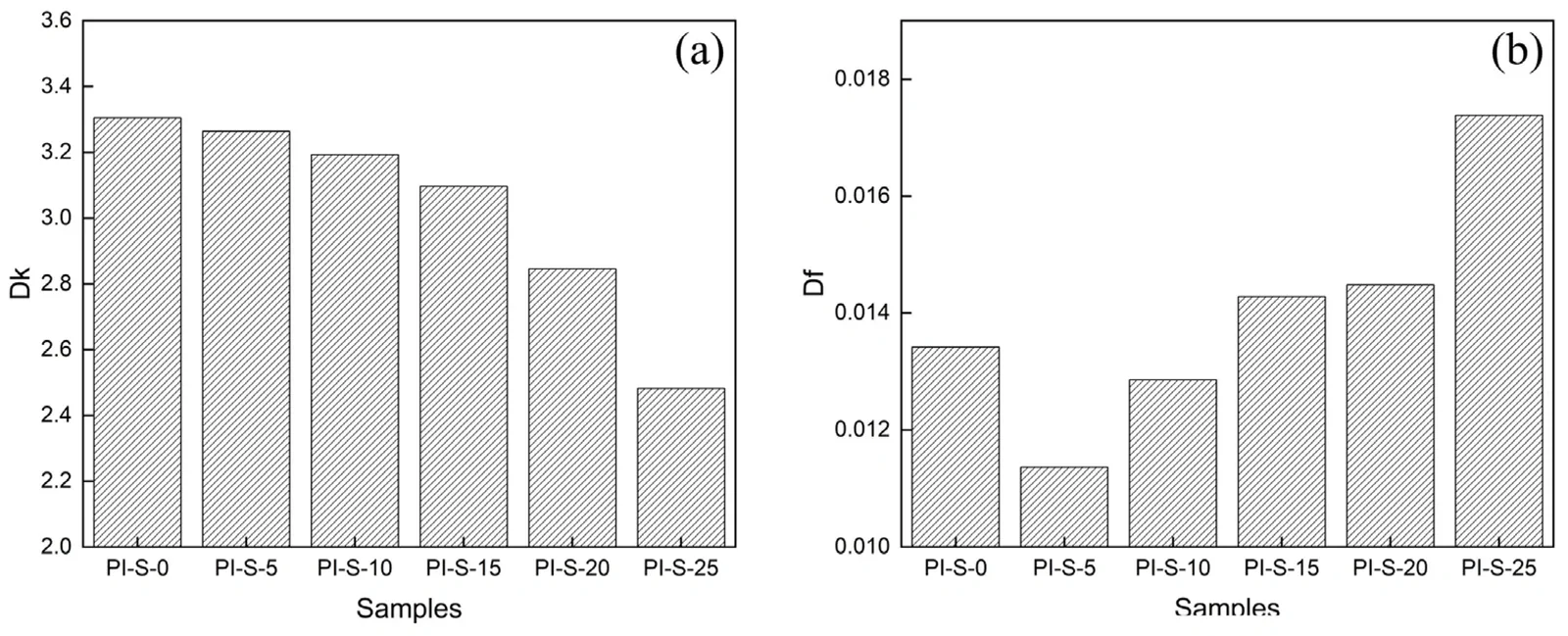
Dielectric Properties Measurement of Films. (**a**) Dielectric constant (Dk) of the films; (**b**) Dissipation factor (Df) of the films.

Figure 4a presents the relative dielectric (Dk) constant of composite films with different hollow SiO_2_ doping ratios. The dielectric constant values are 3.3046 for PI-S-0; 3.2635 for PI-S-5; 3.1926 for PI-S-10; 3.0965 for PI-S-15; 2.8452 for PI-S-20; and 2.4821 for PI-S-25, respectively. Figure 4b shows the corresponding dielectric dissipation factor (Df). The Df values are 0.013415, 0.011363, 0.01285625, 0.0142805, 0.01447975 and 0.017380 for PI-S-0, PI-S-5, PI-S-10, PI-S-15, PI-S-20 and PI-S-25, respectively. Detailed data from replicate measurements are summarized in Table S5 of the Supporting Information.

As shown in Figure 4a, the relative dielectric constant of PI-S composite films decreases monotonically with the increasing doping content of hollow SiO_2_. The decline of dielectric constant is gentle when the doping content ranges from 0% to 5%, and the decreasing magnitude gradually rises from 5% to 25%. PI-S-25 possesses the lowest dielectric constant, which is reduced by approximately 25% in comparison with PI-S-0. It is verified that the introduction of hollow SiO_2_ can effectively lower the dielectric constant of PI films, and the reduction extent is positively correlated with the doping content. From Figure 4b, the dielectric dissipation factor (Df) exhibits a distinct trend: it first decreases and then increases as hollow SiO_2_ loading increases. PI-S-5 achieves the minimum Df value of 0.011363, followed by PI-S-10 with the second-lowest Df of 0.012856.

Among all composite films, PI-S-10 exhibits the optimal thermal stability, and its weight loss rate is close to that of pure PI after 400 °C. Oxidative crosslinking reactions occur in the PI matrix at 50–300 °C. The active sites on the film surface adsorb oxygen molecules and generate oxygen-containing functional groups such as carbonyl and hydroxyl groups, thereby leading to an increase in sample mass. PI-S-0, PI-S-5, PI-S-10 and PI-S-25 show an obvious mass gain before 545 °C. When the temperature reaches 545 °C, all samples enter the rapid thermal decomposition stage, accompanied by a substantial mass loss. The incorporation of hollow SiO_2_ filler impairs the intrinsic thermal stability of the PI matrix to a certain extent.

### 2.5. Thermal Stability Analysis

**Figure 5:**
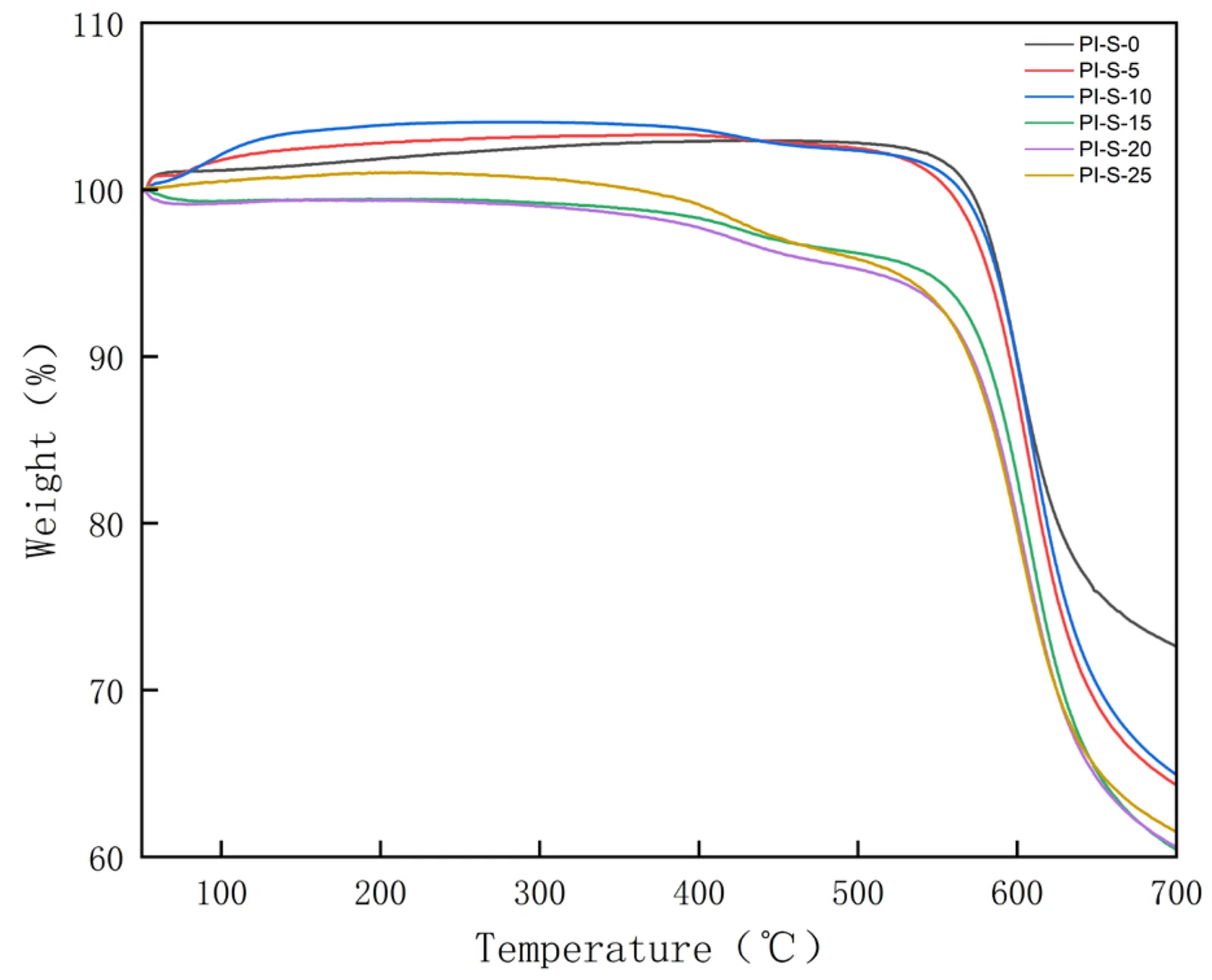
Film thermogravimetric loss curve.

Figure 5 shows the thermogravimetric (TG) curves of the films.

Table 1 lists the weight loss parameters of the composite films. For pristine PI-S-0, the temperature at 5% weight loss is 580.6 °C, the temperature at 10% weight loss is 594.3 °C, and the weight loss ratio at 700 °C is 29.5%. These three indexes are the highest among all samples, demonstrating its optimal thermal stability.

For PI-S-10, 5%loss and 10%loss are 577.3 °C and 591 °C, respectively, with a weight loss of 37.6% at 700 °C. PI-S-10 exhibits the best thermal stability among all doped composites. Mild crosslinking is formed between hollow SiO_2_ and the PI matrix, which effectively restrains the early thermal decomposition process.

Nevertheless, when the doping content increases to 15% and beyond, the thermal stability deteriorates remarkably. The initial decomposition temperature decreases sharply and the carbon residue rate is further reduced. Severe particle agglomeration gives rise to interfacial defects, which consequently advance the thermal decomposition of the composite films.

## 3. Materials and Methods

### 3.1. Film Preparation

All reagents and raw materials were used as received without further purification unless otherwise specified. Pyromellitic dianhydride (PMDA) and poly(amic acid) (PAA) solution derived from 4,4′-oxydianiline (ODA) were purchased from Nantong Bolian Materials Technology Co., Ltd. (Nantong, China). The hollow silica (SiO_2_) nanoparticle solution with a mean particle size of 110 nm was also provided by Nantong Bolian Materials Technology Co., Ltd.

**Figure 6:**
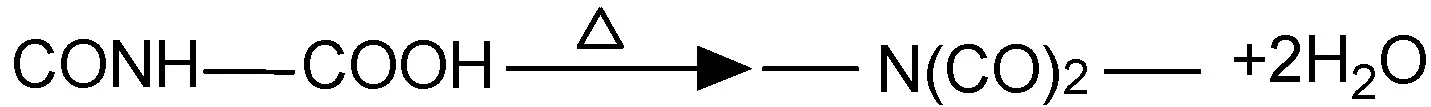
Reaction formula of a single amide acid unit.

Polyimide/SiO_2_ composite films with different hollow SiO_2_ doping ratios were prepared via an in-situ doping method followed by thermal imidization. Briefly, hollow SiO_2_ solution with mass fractions of 0, 5, 10, 15, 20, and 25% was gradually added to the PAA solution under magnetic stirring to form homogeneous PAA- SiO_2_ (PAA-S) mixtures. The resulting mixtures were uniformly coated on the inner wall of circular quartz containers, which were then placed on a three-roll ball mill and processed for 30 min to ensure even distribution of the coating. The coated containers were subsequently transferred to an oven for staged thermal curing: heating at 120 °C for 30 min, then ramping to 160 °C and holding for 30 min, and finally heating to 260 °C and maintaining for 30 min. The heating ramp rate for polyimide was 4 °C/min. The specific reactions occurring in a single poly (amic acid) (PAA) unit at elevated temperatures are illustrated in Figure 6.

**Figure 7:**
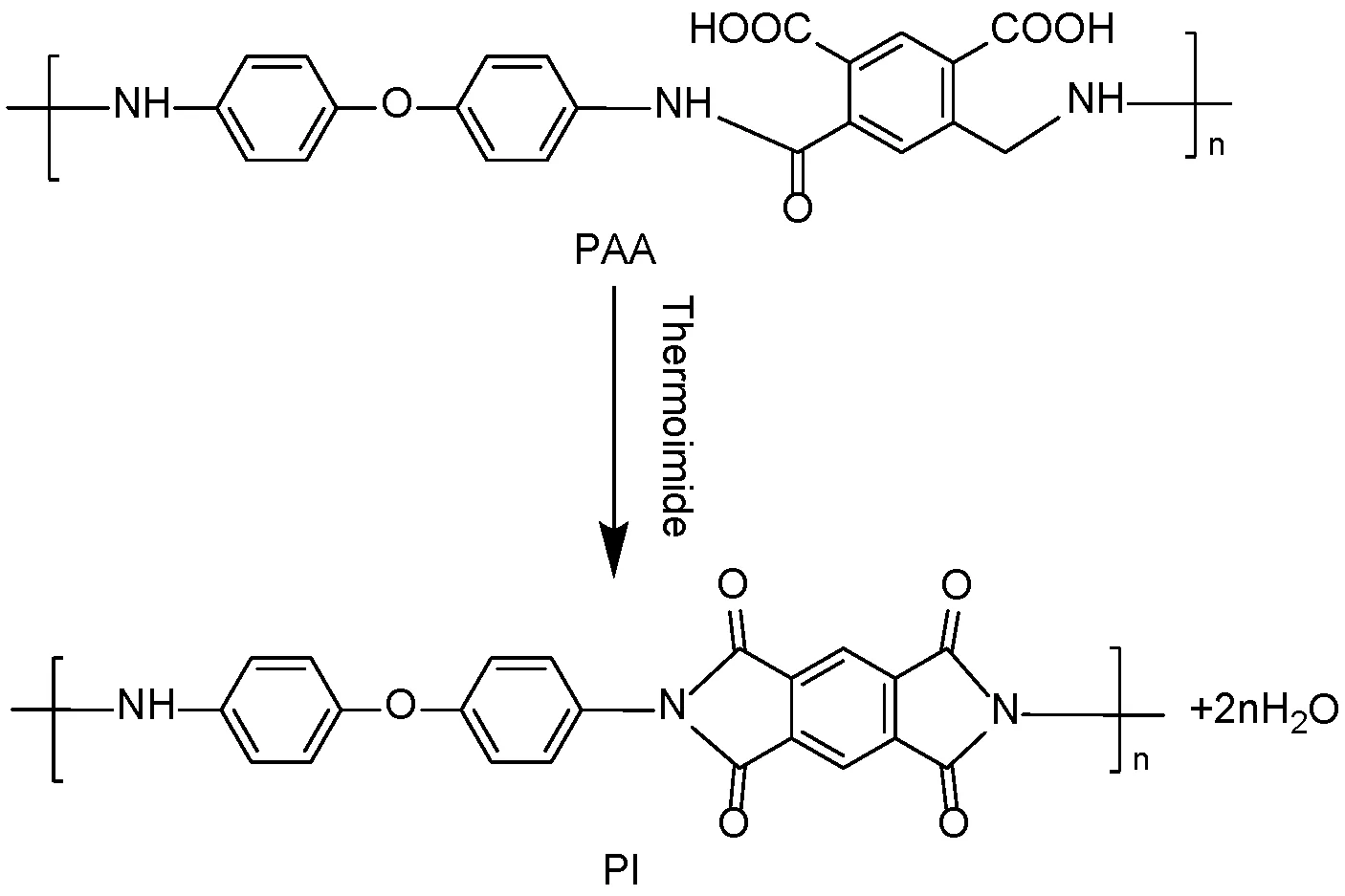
Amide acid solution imidization to form polyimide.

As the reactions of individual poly (amic acid) (PAA) units proceed, the PAA solution begins to undergo thermal imidization, as shown in Figure 7. This thermal imidization process ultimately yields the final PI film.

**Figure 8:**
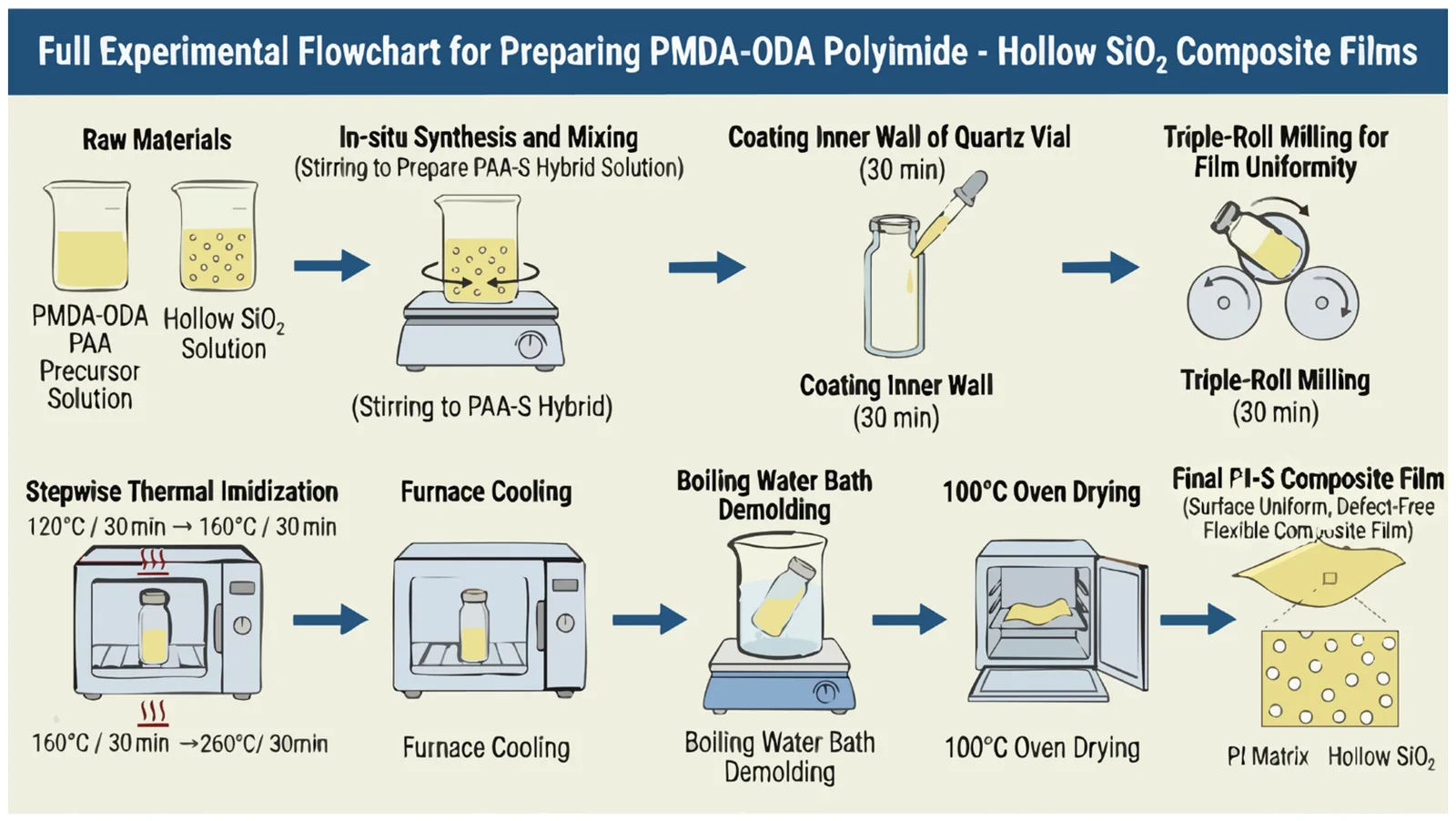
Schematic Diagram of Film Preparation Process.

The oven remained closed throughout the continuous heating process without removing the samples. After curing, the oven was turned off, and the films were naturally cooled to room temperature with the oven door slightly open. The quartz containers were then immersed in boiling water (100 °C) for 20 min. After partial demolding with tweezers, the films were re-immersed in boiling water for an additional 10 min to completely peel off from the containers. The demolded films were dried in an oven at 100 °C for 20 min to remove residual moisture, yielding PI-S composite films with a thickness range of 16–24 μm. The detailed film preparation procedure is illustrated in Figure 8.

The films were labeled as PI-S-0 (pure PI, 0% SiO_2_), PI-S-5 (5% SiO_2_), PI-S-10 (10% SiO_2_), PI-S-15 (15% SiO_2_), PI-S-20 (20% SiO_2_), and PI-S-25 (25% SiO_2_), respectively, and stored in sealed bags at room temperature prior to testing.

### 3.2. Characterization and Performance Measurements

#### 3.2.1. FTIR

Fourier-transform infrared (FTIR) spectroscopy was performed using a Nicolet IS50 spectrometer (Thermo Fisher Scientific, Waltham, MA, USA) to characterize the chemical structure of the films. The spectra were recorded over the wavenumber range of 600–4000 cm^−1^ with a resolution of 2 cm^−1^ and 32 scans per sample.

#### 3.2.2. Mechanical Tensile Test

Mechanical tensile tests were conducted using an HZ-1007E universal tensile testing machine (Dongguan Lixian Instrument Co., Ltd., Dongguan, China). The films were cut into standard dumbbell-shaped specimens with dimensions of 115 mm in total length, 25 mm in width at the grip sections, and 5 mm in the narrow test section. The tensile rate was set to 50 mm/min, and the tensile strength and elongation at break were recorded during the tests.

#### 3.2.3. Electron Microscope

The surface morphology of the composite films was observed using a Nova Nano SEM230 field-emission scanning electron microscope (FEI, Hillsboro, OR, USA). Transmission electron microscopy (TEM) analysis of the hollow SiO_2_ nanoparticles was carried out using a Titan G2 60–300 transmission electron microscope (FEI, Hillsboro, OR, USA).

#### 3.2.4. Dielectric Measurement

The dielectric constant of the films was measured using a Ceyear 3671 split-post dielectric resonator (SPDR, Ceyear Technologies Co., Ltd., Qingdao, China) at a frequency of 10 GHz. The thickness of each film was measured using an AR932 coating thickness gauge (SMART SENSOR, Shenzhen, China) prior to testing.

#### 3.2.5. TGA

Thermogravimetric analysis (TGA) was performed using a TGA1 thermal analyzer (Mettler Toledo, Switzerland) under air atmosphere. The samples were heated from 50 °C to 700 °C at a heating rate of 20 °C/min to evaluate the thermal stability of the composite films.

## 4. Conclusions

In this work, polyimide (PI) nanocomposite films incorporated with 110 nm hollow SiO_2_ nanospheres at weight fractions of 0–25 wt% were fabricated via in-situ filler incorporation followed by thermal imidization. For the first time, the loading-dependent evolution of microstructure, mechanical properties, relative permittivity (εᵣ), and thermal stability in this fixed-particle-size system was systematically evaluated.

The mechanical properties of the nanocomposites exhibit a non-monotonic trend with increasing filler loading, with the optimal comprehensive performance achieved at 10 wt% loading (denoted PI-S-10). At room temperature, PI-S-10 delivers a tensile strength of 68 MPa and 4.5% elongation at break, both markedly enhanced relative to pristine PI, while retaining favorable flexibility and superior mechanical retention after aging at 200 °C and 250 °C. The relative permittivity of the films decreases monotonically with rising SiO_2_ content, from 3.30 for pristine PI (10 GHz) to 2.48 at 25 wt% loading (a 25% reduction). Given the mechanical degradation at high loadings, PI-S-10 (εᵣ = 3.19) is identified as the optimal formulation, striking a well-balanced trade-off between low permittivity and mechanical reliability. Thermogravimetric analysis confirms that pristine PI possesses the highest thermal stability (T_5_% = 580.6 °C), while PI-S-10 shows the best thermal stability among all composites (T_5_% = 577.3 °C), comparable to the pristine matrix. This phenomenon is ascribed to moderate interfacial cross-linking between SiO_2_ fillers and the PI matrix, which suppresses premature thermal decomposition, whereas excessive loading undermines the matrix’s intrinsic thermal stability.

This work demonstrates that in-situ incorporation of size-controlled hollow SiO_2_ nanospheres is an effective strategy to synergistically tailor the multifunctional properties of PI films. The optimized PI-S-10 nanocomposite holds great promise for applications in high-frequency flexible printed circuit boards and microelectronic packaging. Future efforts will focus on interfacial modification and dispersion optimization to further improve the comprehensive performance of PI-based nanocomposites.

## Figures and Tables

**Table 1:** Film weight loss rate.

Samples	5%Loss (°C)	10%Loss (°C)	700 °Closs (%)
PI-S-0	580.6	594.3	29.5%
PI-S-5	569.3	588	37.7%
PI-S-10	577.3	591	37.6%
PI-S-15	551.6	582.3	39.3%
PI-S-20	545.3	573.6	39%

## Data Availability

The data that support the findings are available from the corresponding author upon reasonable request.

## Supplementary Materials

The following supporting information can be downloaded at https://www.mdpi.com/article/10.3390/molecules31193567/s1: Figure S1: Weight Loss Percentages and Curing Degrees of PI-x Films: (a) weight loss percentage; (b) curing degree; Table S1: Sample Designations for Specimens at Different Curing Temperatures; Table S2: Imidization Degree of PI-x Films; Table S3: Tensile strength test data; Table S4: Elongation at break test data; Table S5: The specific values of dielectric constant and dielectric dissipation factor.

## Abbreviations

The following abbreviations are used in this manuscript:

PI	polyimide
PAA	polyamic acid
SiO_2_	Silicon Dioxide

## References

[1] Namathoti S., Sahu S.K., Sahu D., Shanmugam R., Sreekanth P.S.R. (2026). Evaluation of shape recovery stress and motion derivatives in 4D printed shape memory polymers for actuator application. Int. J. Polym. Anal. Charact..

[2] Geiß P.L., Schumann M. (2026). Gelation, Vitrification and Shrinkage of Thermoset Polymers—Methods for Investigation and Modelling. Mater. Sci. Forum.

[3] Hu L.Q., Jiao Y., Zhang G.Y., Guan J.Y., Bui V.T., Lin H.Q. (2026). Polymer-derived carbon molecular sieves with tailored polymodal pores for membrane separations. Prog. Polym. Sci..

[4] Shi K.L., Chen H.P., Li J.W., Han Y.P., Sun H.L., Liu H., Dong L., Liu C.T., Shen C.Y. (2026). Versatile fully polymeric conductive organohydrogel as an all-in-one wearable response platform for bioelectronics. Chem. Eng. J..

[5] Wu F., Misra M., Mohanty A.K. (2021). Challenges and new opportunities on barrier performance of biodegradable polymers for sustainable packaging. Prog. Polym. Sci..

[6] Ye J., Xu A., Wang Z.J., Luo H., Yang X.W., Chen S. (2026). Strategic structural design of polyimide dielectrics toward superior high-temperature energy storage performance. Compos. Sci. Technol..

[7] Tang Z.Y., Zhang X., Su L.H., You P.J., Li X., Fan W., Liu T.X. (2026). Ablation-resistant shape memory polyimide composite aerogels with exceptional high temperature response for smart thermal protection. Compos. Part A-Appl. Sci. Manuf..

[8] He X., Ma S., Jiang J., Hua Z.B., Liu L., Huang Y.D. (2026). A high temperature shape memory polyimide with high transparency and intrinsic low dielectric constant. Polymer.

[9] Dong X.D., Wan B.Q., Zha J.W. (2024). Versatile Landscape of Low-k Polyimide: Theories, Synthesis, Synergistic Properties, and Industrial Integration. Chem. Rev..

[10] Li Y.H., Sun G.H., Zhou Y., Liu G.M., Wang J., Han S.H. (2022). Progress in low dielectric polyimide film—A review. Prog. Org. Coat..

[11] Liu X.J., Zheng M.S., Chen G., Dang Z.M., Zha J.W. (2022). High-temperature polyimide dielectric materials for energy storage: Theory, design, preparation and properties. Energy Environ. Sci..

[12] Wei B.J., Luo W.M., Du J.Y., Ding Y.F., Guo Y.J., Zhu G.M., Zhu Y., Li B.W. (2024). Thermal interface materials: From fundamental research to applications. Susmat.

[13] Beltran V.V., Wang R., Hong J.Y., Jung Y., Moon S., Kwon D.K., Lee J.K. (2026). Enhancing the electric conductivity and surface smoothness of photosintered copper films on polyimide substrates. Addit. Manuf..

[14] Zhu L.K., Yuan P.M., Kou X.Y., Jing B.Q., An W., Ma R., Hu D.W. (2025). Fluorinated polyimide/barium titanate@polyimide/fluorinated polyimide sandwich nanocomposite film capacitors for high-temperature energy storage and piezoelectric sensing. Colloids Surf. A-Physicochem. Eng. Asp..

[15] Zhang Z.L., Xu Y.H., Huang C.Y., Zhao Y., Zhu J., Shan F. (2025). A Promising Passivation Strategy Using Polyimide for Enhancing the Performance of Metal Oxide Thin-Film Transistors. J. Electron. Mater..

[16] Peng K., Yang X.Y., Islam M.Z., Mei X.Y., Jiang Y.H., Qi X.M., Fu Y.Q. (2024). The Sandwich Structure of a Polyimide/PTFE Composite Film Designed to Achieve a Low Dielectric Constant and Excellent Thermal Performance at High Frequency. Acs Appl. Polym. Mater..

[17] Zhang Y., Wang J.W., Ma Y.J., Zhang Z.L. (2024). Multifunctional boron nitride nanosheets/fluorinated-polyimide composites with ultra-low dielectric constant and high thermal conductivity. Polym. Compos..

[18] Zhang Z., Dong W., Zou H.M., Zhao S., Chai C.Z., Shi Y.S., Lv X.J., Shu Q.H. (2024). Polyimide-based high-temperature-resistant films for electromagnetic-interference shielding. Surf. Interfaces.

[19] Shao Z.J., Liu Y.N., Yang D., Li L.F., Xia Y., Zhang L.H., Tong B., Wang B.J., Zhao Y., Xiao Z. (2024). Polyimide Enables Carbon-Based Conductive Polymer Composites with High Working Temperature for Deicing Application. J. Electron. Mater..

[20] Wang H.Q., Zhang J.L., Li J.H., Lv X.L., Zhang S.Y., Zhang G.P., Sun R. (2024). A strategy for the preparation of low dielectric FGQD/PSPI composite films in wafer-level packaging. Mater. Today Commun..

[21] Si J.J., Ono H., Uchida K., Nozaki S., Morisaki H., Itoh N. (2001). Correlation between the dielectric constant and porosity of nanoporous silica thin films deposited by the gas evaporation technique. Appl. Phys. Lett..

[22] Abdullaev A., Liu F.J., Peng K., You Y.J., Qu K.H., Islam Z., Si Y.S., Fu Y.Q. (2025). Production of PI/PTFE@PI nanocomposite membranes as dielectric materials using electrospinning and dip-coating methods. Compos. Commun..

[23] Abdullaev A., Mei X.Y., Liu F.J., Peng K., You Y.J., Wu F.Y., Islam M.Z., Yang Q.B., Si Y.S., Fu Y.Q. (2024). Superhydrophobic Electrospun PI/PTFE Membranes with Ultralow Dielectric Constants for High-Frequency Telecommunication. Acs Appl. Mater. Interfaces.

[24] Zhang Y.H., Lu S.G., Li Y.Q., Dang Z.M., Xin J.H., Fu S.Y., Li G.T., Guo R.R., Li L.F. (2005). Novel Silica Tube/Polyimide Composite Films with Variable Low Dielectric Constant. Adv. Mater..

