# Supplementary material for: Synergistically Enhanced Properties of Polyimide Films via In-Situ Hollow SiO2 Nanospheres
Source: Molecules. 2026 Oct 7;31(19):3567. doi: 10.3390/molecules31193567 (PMC13666769; doi:10.3390/molecules31193567)
Supplement: Supplementary file 1 [file molecules-31-03567-s001.zip › molecules-4473090-supplementary.pdf]

Supporting information

Synergistically Enhanced Properties of Polyimide Films via In-Situ Hollow SiO_2 Nanospheres

Zheng Wu [1,2], Rui Meng [1], Chonggui Zhong [1], Yanyan Huang [1], Juehong Guo [2], Yuchi Cheng [1,*] and Zhongwei Yu [1,*]

1 School of Physical Science and Technology, Nantong University, Nantong 226019, China; wuz@boliantech.com (Z.W.); 17851870775@163.com (R.M.); chgzhong@ntu.edu.cn (C.Z.); yyhuang@ntu.edu.cn (Y.H.)
2 Nantong Bolian Tech Co., Ltd., Nantong 226019, China; tech@boliantech.com
* Correspondence: chengyc@ntu.edu.cn (Y.C.); yu.zw@ntu.edu.cn (Z.Y.)

Determination of Imidization Degree of PI Films

Prior to fabricating composite films, the fabrication procedure for neat polyimide (PI) films must be confirmed. The imidization degree acts as a core indicator for evaluating the completeness of the polycondensation reaction from poly(amic acid) (PAA) to PI. A higher degree of curing indicates more thorough polycondensation of PAA, which yields PI films with superior comprehensive properties.

After specimen coating, a stepwise heating curing program was implemented. The thermal procedure consisted of sequential thermal holding stages: 120 °C for 30 min, followed by 160 °C for another 30 min, and terminal heat treatment at 180 °C, 200 °C, 220 °C, 240 °C and 260 °C (30 min holding for each terminal temperature), respectively. Five batches of films with different terminal temperature gradients were fabricated accordingly. Polyimide films cured with the final heating temperature of 180 °C were labeled PI-1, and the rest of the films were numbered following the same rule, as shown in Table S1.

Table S1. Sample Designations for Specimens at Different Curing Temperatures.

Temperature			Samples
120°C	180°C	180°C	PI-1
		200°C	PI-2
		220°C	PI-3
		240°C	PI-4
		260°C	PI-5

The swelling method was utilized to characterize the degree of curing of polyimide (PI) films prepared under diverse heat treatment procedures, thereby screening the optimal heat treatment temperature. The procedure used is defined as follows:

1. All test films were cut into standard 10 cm × 10 cm specimens and weighed; the recorded mass was denoted as M_1.

2. A volume of 100 mL N-methylpyrrolidone (NMP) solvent was poured into each swelling vessel to guarantee the full submersion of the film samples.

3. All vessels were sealed and transferred to a constant-temperature oven for swelling at 100 °C over 10 h.

4. Upon completion of the swelling procedure, the specimens were removed using tweezers and soaked in boiling deionized water for 5 min to wash off residual solvent attached to sample surfaces.

5. The pretreated samples were placed in a constant-temperature oven at 100 °C for 20 min, followed by baking at 180 °C for an additional 5 min to thoroughly eliminate residual water and trace solvent trapped within the films.

6. The dried specimens were taken out, cooled to ambient temperature, and weighed once more, with the acquired mass marked as M_2.

Subsequently, the measured masses were substituted into the following formula:

$$weight\ loss = \frac{M_1 - M_2}{M_1} \times 100\% \quad (1)$$

$$curing\ degree = 100\% - weight\ loss \quad (2)$$

The corresponding data are summarized in Table S2.

Table S2. Imidization Degree of PI-x Films.

Samples	FFT（℃）	m1（mg）	m2（mg）	WL（%）	C（%）
PI-1	180	47.57	44.65	6.13	93.87
PI-2	200	48.24	45.90	4.87	95.13
PI-3	220	47.32	46.48	1.78	98.22
PI-4	240	48.54	47.89	1.34	98.66
PI-5	260	48.46	48.26	0.41	99.59

FFT: Film-forming Temperature, defined as the temperature at which films are preliminarily coagulated from PAA solution; WL: Weight Loss; C: Curing Degree.

Figure S1 illustrates the weight loss percentages and curing degrees of PI-x films. Subplot (a) corresponds to the weight loss percentages of PI-x samples, while subplot (b) presents their curing degrees. As observed in the figure, the curing degree of PI films shows a positive correlation with heat treatment temperature. The PI-2 film thermally cured at 200 °C attains a curing degree of 95%, reflecting a satisfactory curing performance. Furthermore, the curing degree of PI-5 reaches 99%, which verifies that the full imidization of PI films can be accomplished at 260 °C.

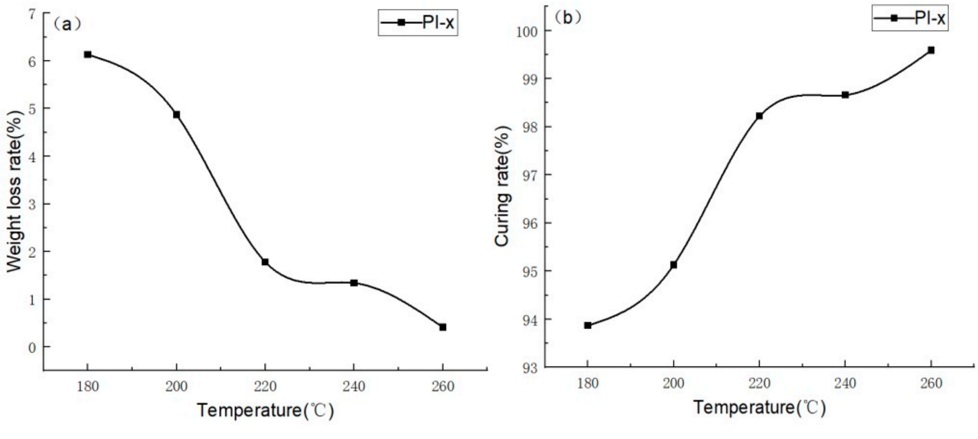

Figure S1. Weight Loss Percentages and Curing Degrees of PI-x Films: (a) Weight loss percentage; (b) Curing degree.

Accordingly, the optimal fabrication protocol of PI films for this study was established. Composite films were manufactured using a stepwise thermal treatment procedure: thermal holding

at 120 °C for 30 min, subsequent holding at 160 °C for 30 min, and a final thermal holding step at 260 °C for 30 min.

Summary of mechanical properties test data

Table S3. Tensile strength test data.

Samples	Temperature conditions (°C)	Values of three tests (MPa)			Mean (MPa)
PI-S-0	room temperature	51.10	53.49	48.71	51.10
	200	47.64	57.51	44.14	49.76
	250	36.14	39.11	46.43	40.56
PI-S-5	room temperature	60.42	65.28	61.14	62.28
	200	62.28	56.47	60.93	59.89
	250	45.50	57.11	42.91	48.53
PI-S-10	room temperature	71.45	68.49	63.85	67.93
	200	65.86	63.07	63.71	64.21
	250	62.75	67.92	68.11	66.26
PI-S-15	room temperature	70.71	49.00	53.21	57.64
	200	53.29	49.86	52.43	51.86
	250	57.59	60.24	52.51	56.78
PI-S-20	room temperature	67.24	61.32	70.1	66.22
	200	54.64	62.36	56.71	57.90
	250	66.88	61.89	58.55	62.44
PI-S-25	room temperature	49.93	58.79	61.41	56.71
	200	56.71	55.79	55.36	55.95
	250	50.49	51.40	52.31	51.40

Room temperature: 25 °C.

Table S4. Elongation at break test data.

Samples	Temperature conditions (°C)	Values of three tests (%)			Mean (%)
PI-S-0	room temperature	2.23	2.70	2.21	2.38
	200	2.15	2.63	1.89	2.22
	250	1.75	2.11	1.98	1.95
PI-S-5	room temperature	4.31	4.08	4.13	4.17
	200	3.16	2.72	3.13	3.00
	250	2.14	2.19	2.24	2.19
PI-S-10	room temperature	4.76	4.34	4.21	4.44
	200	3.29	3.33	3.26	3.29
	250	2.69	3.26	2.44	2.80
PI-S-15	room temperature	2.72	2.56	2.1	2.46
	200	1.9	2.12	2.48	2.13
	250	1.86	2.21	1.66	1.91
PI-S-20	room temperature	2.35	2.41	2.45	2.40
	200	2.25	2.02	2.01	2.09
	250	1.74	2.17	2.13	2.01
PI-S-25	room temperature	2.33	2.93	2.81	2.69
	200	2.21	2.14	2.65	2.33
	250	2.45	2.29	1.91	2.22

Room temperature: 25 °C.

Summary of dielectric test values

Table S5. The specific values of dielectric constant and dielectric dissipation factor.

Samples	Film thickness	Dielectric constant	Mean	Dielectric loss	Mean
PI-S-0	17	3.3248	3.3046	0.013098	0.013415
	17	3.338		0.013195	
	17	3.2689		0.013651	
	17	3.2865		0.013717	
PI-S-5	17	3.2514	3.2635	0.011242	0.011363
	17	3.2676		0.011182	
	18	3.2594		0.011384	
	18	3.2756		0.011644	
PI-S-10	18	3.4667	3.1926	0.013252	0.01285625
	18	3.475		0.013088	
	20	2.9093		0.012523	
	20	2.9194		0.012562	
PI-S-15	23	2.9823	3.0965	0.013976	0.0142805
	23	2.9898		0.014084	
	25	3.2031		0.014507	
	25	3.2109		0.014555	
PI-S-20	20	2.8831	2.8452	0.014602	0.01447975
	20	2.8909		0.014519	
	22	2.8002		0.014367	
	22	2.8067		0.014431	
PI-S-25	22	2.5688	2.4821	0.016715	0.017380
	22	2.5916		0.016739	
	24	2.384		0.018049	
	24	2.384		0.018017	

Four replicate tests per sample.
